# Immune Checkpoint Inhibitor and Radiotherapy-Related Pneumonitis: An Informatics Approach to Determine Real-World Incidence, Severity, Management, and Resource Implications

**DOI:** 10.3389/fmed.2021.764563

**Published:** 2021-11-01

**Authors:** Sumeet Hindocha, Des Campbell, Merina Ahmed, Kyriaki Giorgakoudi, Bhupinder Sharma, Nadia Yousaf, Philip Molyneaux, Benjamin Hunter, Hardeep Kalsi, Wanyuan Cui, Michael Davidson, Jaishree Bhosle, Anna Minchom, Imogen Locke, Fiona McDonald, Mary O'Brien, Sanjay Popat, Richard W. Lee

**Affiliations:** ^1^Lung Unit, The Royal Marsden, National Health Service (NHS) Foundation Trust, London, United Kingdom; ^2^Artificial Intelligence (AI) for Healthcare Centre for Doctoral Training, Imperial CollegeLondon, London, United Kingdom; ^3^Early Diagnosis and Detection, The National Institute for Health Research (NIHR) Biomedical Research Centre at The Royal Marsden NHS Foundation Trust and The Institute of Cancer Research, London, United Kingdom; ^4^Performance & Information Department, The Royal Marsden, National Health Service (NHS) Foundation Trust, London, United Kingdom; ^5^School of Health Sciences, City University of London, London, United Kingdom; ^6^Radiology Department, The Royal Marsden, National Health Service (NHS) Foundation Trust, London, United Kingdom; ^7^Fibrosis Research Group, Inflammation, Repair and Development Section, National Heart and Lung Institute, Imperial College London, London, United Kingdom; ^8^Lung Unit, The Royal Marsden, National Health Service (NHS) Foundation Trust, Sutton, United Kingdom

**Keywords:** pneumonitis, immunotherapy, checkpoint inhibitor, radiotherapy, informatics, health economics

## Abstract

Pneumonitis is a well-described, potentially life-threatening adverse effect of immune checkpoint inhibitors (ICI) and thoracic radiotherapy. It can require additional investigations, treatment, and interruption of cancer therapy. It is important for clinicians to have an awareness of its incidence and severity, however real-world data are lacking and do not always correlate with findings from clinical trials. Similarly, there is a dearth of information on cost impact of symptomatic pneumonitis. Informatics approaches are increasingly being applied to healthcare data for their ability to identify specific patient cohorts efficiently, at scale. We developed a Structured Query Language (SQL)-based informatics algorithm which we applied to CT report text to identify cases of ICI and radiotherapy pneumonitis between 1/1/2015 and 31/12/2020. Further data on severity, investigations, medical management were also acquired from the electronic health record. We identified 248 cases of pneumonitis attributable to ICI and/or radiotherapy, of which 139 were symptomatic with CTCAE severity grade 2 or more. The grade ≥2 ICI pneumonitis incidence in our cohort is 5.43%, greater than the all-grade 1.3–2.7% incidence reported in the literature. Time to onset of ICI pneumonitis was also longer in our cohort (mean 4.5 months, range 4 days-21 months), compared to the median 2.7 months (range 9 days−19.2 months) described in the literature. The estimated average healthcare cost of symptomatic pneumonitis is £3932.33 per patient. In this study we use an informatics approach to present new real-world data on the incidence, severity, management, and resource burden of ICI and radiotherapy pneumonitis. To our knowledge, this is the first study to look at real-world incidence and healthcare resource utilisation at the per-patient level in a UK cancer hospital. Improved management of pneumonitis may facilitate prompt continuation of cancer therapy, and improved outcomes for this not insubstantial cohort of patients.

## Introduction

Pneumonitis is a well-described, potentially life-threatening and disabling adverse effect of several cancer therapies including immune checkpoint inhibitor (ICI) drugs and thoracic radiotherapy. The advent of ICI drugs, such as those targeting the Programmed Cell Death 1 receptor or its ligand (PD1/PD-L1), have transformed cancer treatment over the last decade. The numbers of patients receiving such drugs either alone or in combination therapy for various cancers continues to increase and an abundance of clinical trials elaborate upon further applications (1, 2). Radiotherapy continues to be a major treatment modality for lung cancer and with an ageing and increasingly comorbid population, the number of patients treated curatively with radiotherapy rather than surgery is likely to increase. As pneumonitis is observed in up to 5% of patients treated with ICI (3–5) and 40% following radiotherapy (6), it is likely to present a growing problem in cancer care. As pneumonitis can result in significant morbidity, preclusion of further treatment and even death (7), it is important for clinicians to have an awareness of its incidence and severity.

There is a lack of real-world incidence data in the literature, and that which exists does not always correlate with findings from clinical trials (8). Similarly, there is a dearth of information on health resource utilisation by patients with pneumonitis, which is an important element of economic evaluation studies seeking to explore whether new approaches to treatment are cost-effective.

Informatics approaches are increasingly being applied to healthcare data for their ability to identify specific patient cohorts at scale, more efficiently than traditional manual approaches. Here we use informatics tools to assist identification of cases of ICI and radiotherapy-pneumonitis and describe data on clinical management and impact of such cases in our specialist cancer centre from 2015 to 2020 inclusive.

## Materials and Methods

Using our integrated data warehouse and electronic health record (EHR) systems, we developed a Structured Query Language (SQL)-based informatics algorithm which we applied to CT Thorax report text to identify scans performed between 01/01/2015 and 31/12/2020 that contained key terms determined by radiology and respiratory expertise: “pneumonitis,” “pulmonary toxicity,” “lung toxicity,” “lung injury,” “interstitial lung disease” and “pneumonia”. This generated a list of 3,632 CT reports. The report text, scan date, and patient identifiers were extracted along with additional demographic and treatment details.

Such terms were typically located within the “request details” section of the report, where the requesting clinician had queried presence of “pneumonitis,” separately from the radiologist's findings. In order to identify CT reports where the body of the report described findings relating to possible pneumonitis, further rules were applied to filter reports containing the terms “ground glass,” “treatment,” “drug,” “diffuse,” “infiltrates,” “radiotherapy,” “radiation” again guided by respiratory and radiology expertise. This filtered the number of reports down to 2,416.

Philters were also applied to structured treatment fields to identify only patients who had received any of the ICI drugs prescribed at our centre (Atezolizumab, Avelumab, Durvalumab, Ipilimumab, Nivolumab, Pembrolizumab) and/or to identify all patients that had received radiotherapy to the thorax/upper abdomen/neck/vertebrae/breast/chest wall prior to the date of the flagged CT report. A manual validation step was undertaken to eliminate any reports that did not suggest a possible pneumonitis. Reports from additional scans performed within 6 months of the identified scan for each patient were also eliminated, to limit the dataset to the earliest CT with reported pneumonitis.

The EHR of all patients with pneumonitis possibly attributable to ICI and/or RT was manually reviewed to identify the earliest date where potential pneumonitis symptoms (e.g., cough, dyspnoea) or clinical reference to pneumonitis began, and to ascertain severity of pneumonitis, which was inferred according to CTCAE v5.0 criteria (Supplementary Table 1). A case of pneumonitis was defined as a radiological report consistent with pneumonitis or treating clinician or multidisciplinary meeting consensus of pneumonitis based on the clinical picture at the time of presentation. Pneumonitis was attributed to ICI if one of the above six drugs was administered within the preceding 3 months, and considered radiotherapy-related if the patient was treated with radiation in the preceding 12 months. Fifty cases from the final cohort (20%) were randomly selected for independent review and scoring of CTCAE severity by a second clinician. The Krippendorff's alpha measure of inter-observer correlation (9) was used to check concordance between both clinicians' interpretation of severity, calculated using the Natural Language ToolKit (NLTK) package in Python v 3.9.

Where symptoms were CTCAE grade 2 or more or treatment was withdrawn, additional data including investigations and medical management with steroids and/or antibiotics were collected manually from the EHR. Where a patient had more than one distinct episode of pneumonitis, this was recorded as a separate case. Further data were collected from the pharmacy database on the total number of patients treated with the above-mentioned ICI drugs (as mono- or combination-therapy) between 2015 and 2020 and for all patients deemed to have ICI or radiotherapy-related pneumonitis, clinical activity data was extracted algorithmically from the EHR including total number of consultant follow-up clinic attendances, CT thorax scan appointments and MDT discussions for the 16-weeks after the date of pneumonitis diagnosis.

## Results

### The Cohort

**Figure 1:**
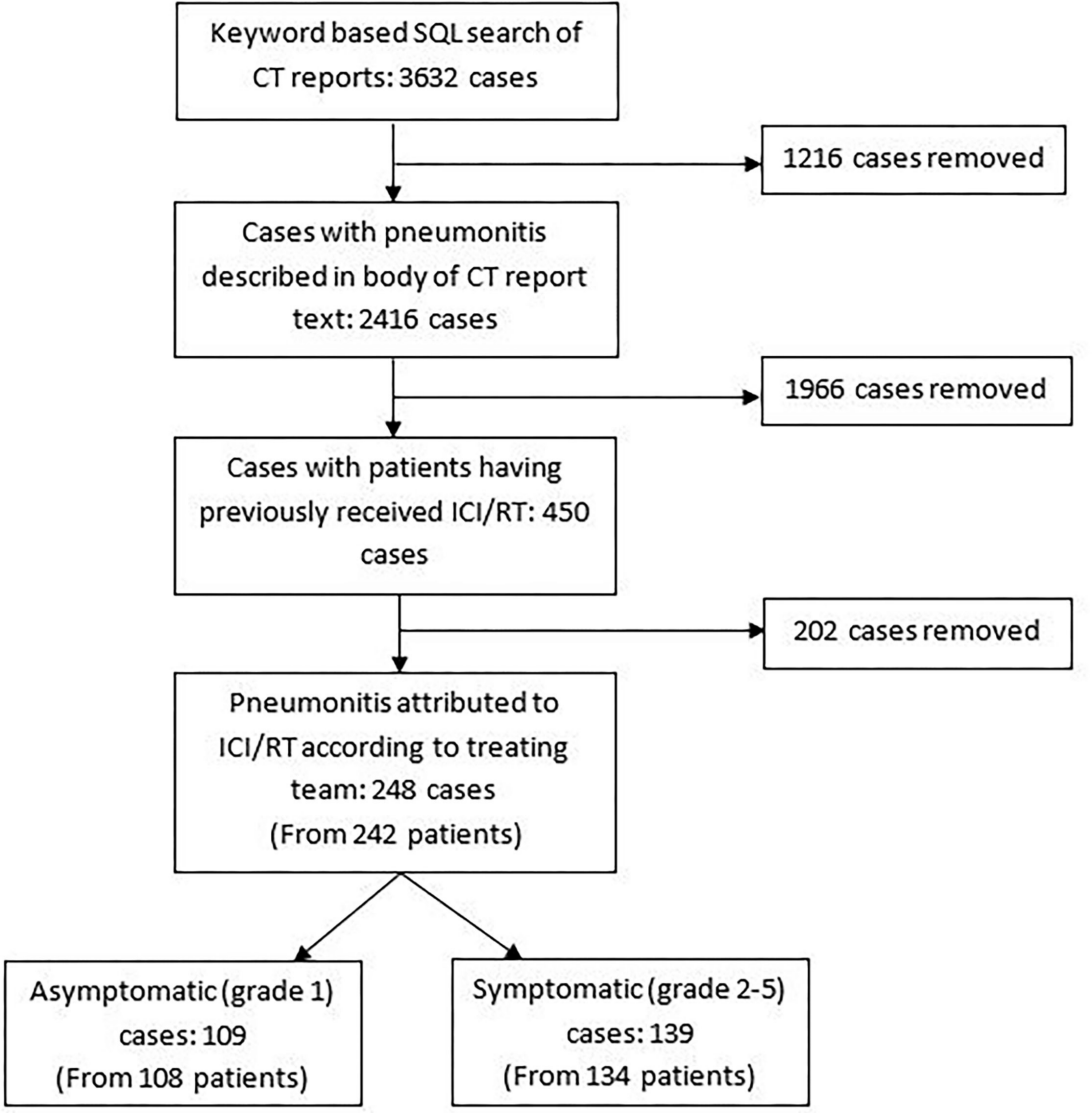
Flow of data to identify cases for inclusion. RT, radiotherapy.

We identified 450 CT reports indicating a pneumonitis in patients who had previously received ICI and/or radiotherapy, and thus which was possibly attributable to ICI/RT amongst other causes (Figure 1). In 248 cases (from 242 patients), the pneumonitis was attributed by the treating team as partially or completely due to ICI and/or radiotherapy-pneumonitis. In some cases, infection and other co-morbidities (e.g., pulmonary embolus or COPD) may have also contributed to the presentation as per the clinical opinion of the treating team at the time; for example due to CT pulmonary angiogram results demonstrating the presence of both pulmonary emboli and features in keeping with pneumonitis, or raised inflammatory markers and pyrexia at presentation which responded to antibiotic therapy. One hundred and nine cases were asymptomatic (grade 1) and 139 were symptomatic with severity grade 2 or more. Results of symptomatic cases are presented below.

Table 1 shows the demographic and aetiology breakdown of symptomatic cases. Of the 139 symptomatic cases, 61% of patients were male, the majority were performance status 0–1 and 20.14% were never-smokers. ICI accounted for 61.15% of cases with 7.19% of cases attributed to a combination of both ICI and radiotherapy.

**Table 1:** Symptomatic (grade ≥2) pneumonitis demographics and aetiology.

**Demographics and causes**	**Number**
Age	Average 66, Range: 27–87
Gender Male Female	85 (61%) 54 (39%)
Performance status 0 1 2 3 No data	20 (14.4%) 89 (64%) 21 (15.1%) 5 (3.6%) 4 (2.9%)
Smoking status Never Ex Current No Data	28 (20.14%) 82 (58.99%) 11 (7.91%) 18 (12.94%)
Aetiology Radiotherapy ICI Radiotherapy and ICI combined	44 (31.65%) 85 (61.15%) 10 (7.19%)

**Figure 2:**
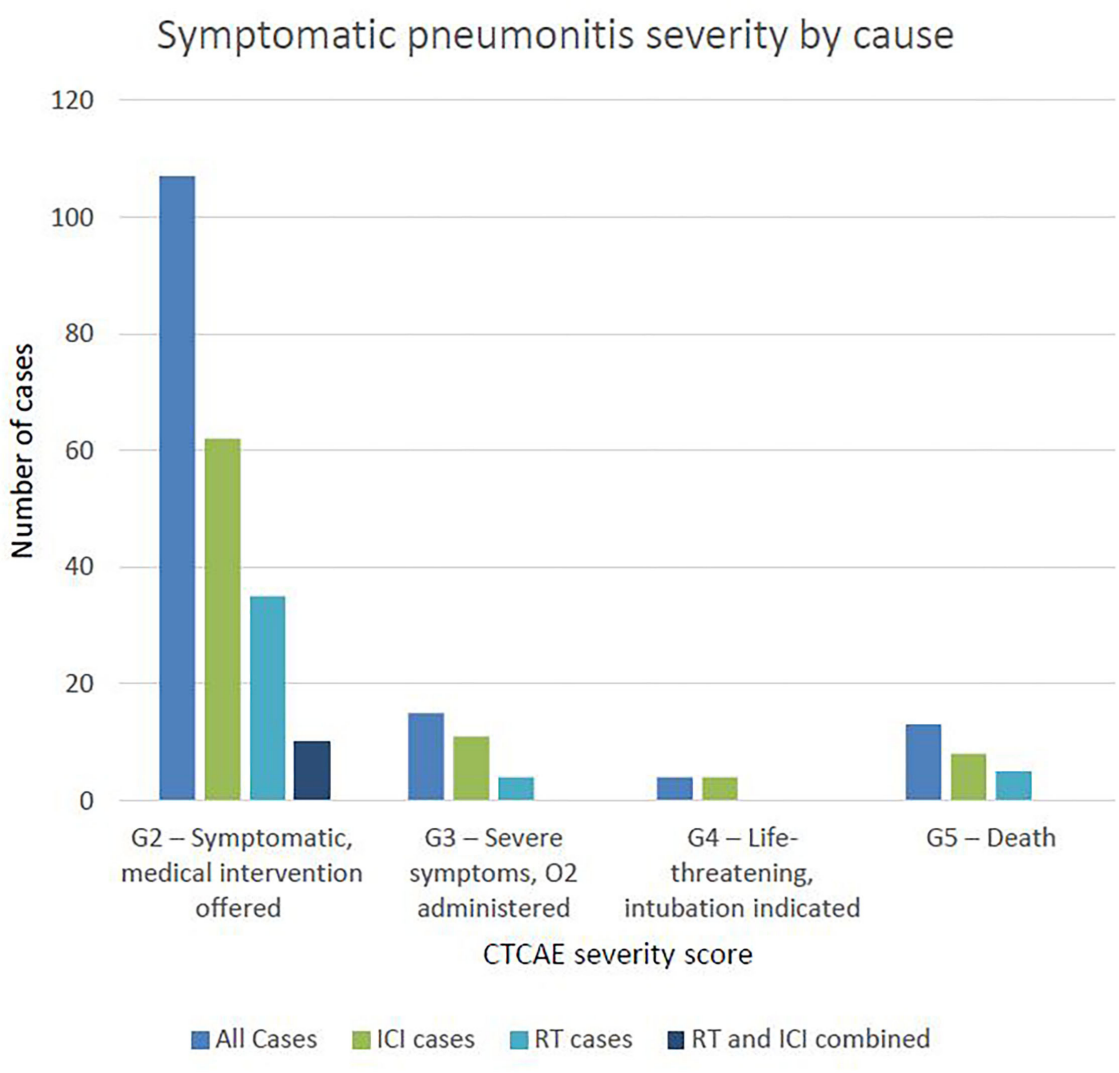
Symptomatic pneumonitis severity by cause.

**Figure 3:**
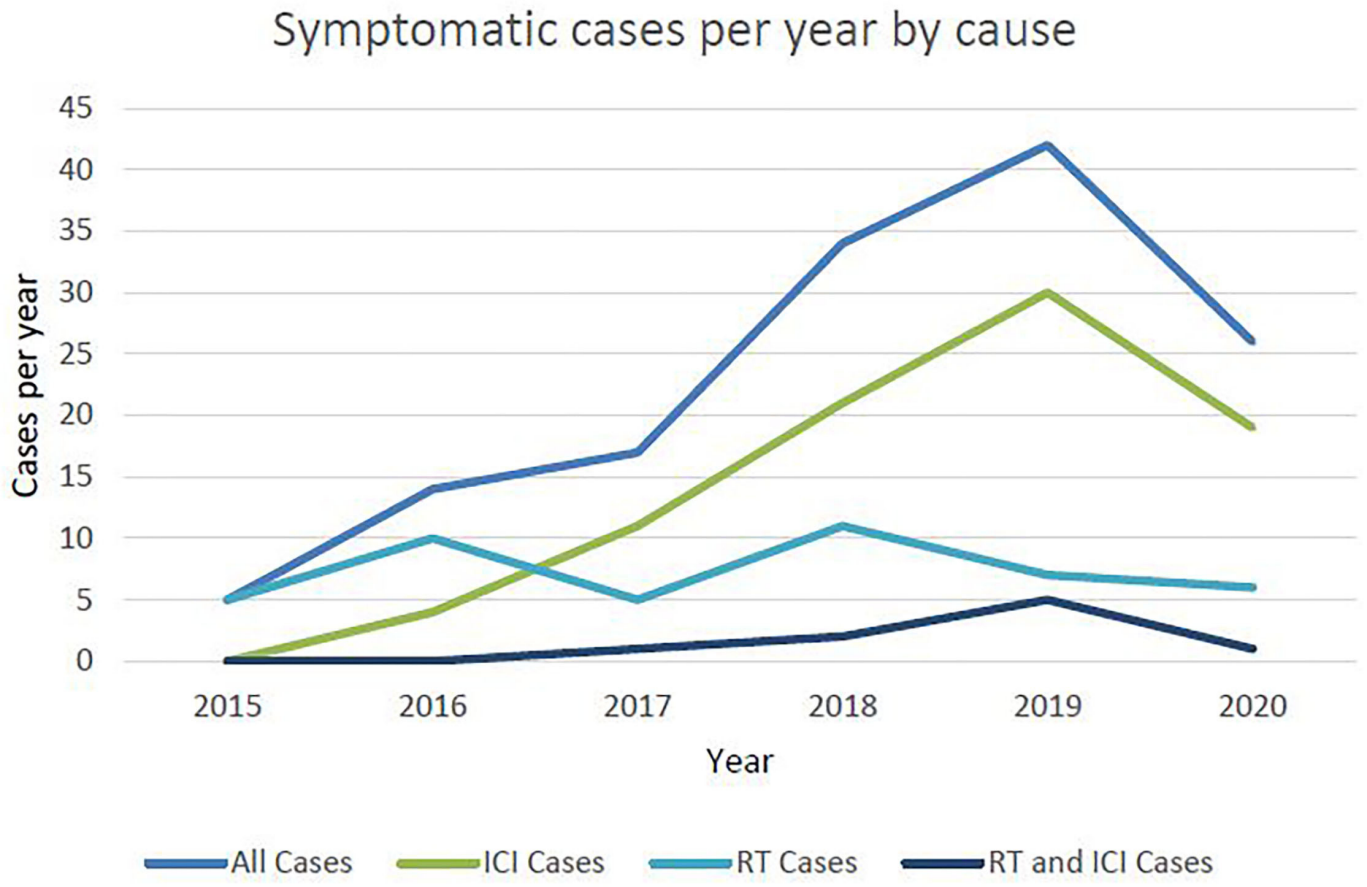
Number of symptomatic cases per year by cause.

Three quarters of cases were of grade 2 severity, 10.79% were grade 3 severity, 4 patients (2.88%) required intubation (grade 4) and 13 patients (9.35%) died (grade 5) (Figure 2). Of those that died, average time from first diagnosis of pneumonitis to death was 64 days (range 6–378). The number of cases increased year-on-year until 2020 after which there was a marked reduction (Figure 3). For the 50 cases where a second clinician independently reviewed and scored CTCAE severity, the Krippendorff's alpha measure of inter-observer correlation demonstrated high concordance at 0.97.

One hundred and thirty-four symptomatic cases (96.4%) were treated with intravenous or oral steroids (Table 2). Two patients declined, 1 was treated with a steroid inhaler and in one grade 2 case, steroids were withheld due to concerns of possible COVID-19 and on-going tumour response to immunotherapy. A third of cases treated with oral steroids were given prophylactic co-trimoxazole at the time of steroid initiation, and this was mostly in cases where pneumonitis was described in the EHR to be ICI-related. Seventy-nine cases (57%) were treated with empirical antibiotics at initial presentation. Six cases (all ICI-pneumonitis) went on to receive another immunosuppressive therapy (Infliximab or Mycophenolate Mofetil).

**Table 2:** Medical treatment, investigation, and management for symptomatic pneumonitis.

**Medical treatment**	**All cases** **(*n* = 139)**	**ICI cases** **(*n* = 85)**	**RT cases** **(*n* = 44)**	**RT and ICI cases** **(*n* = 10)**
Empirical antibiotics	79 (56.8%)	49 (57.6%)	25 (56.8%)	5 (50%)
Steroids (oral or intravenous)	134 (96.4%)	84 (98.8%)	40 (90.9%)	10 (100%)
Prophylactic co-trimoxazole at initiation of oral steroids	41 (29.5%)	37 (43.5%)	1 (2.3%)	3 (30%)
Other immunosuppressive agent	6 (4.3%)	6 (7.1%)	0	0
**INVESTIGATION AND MANAGEMENT**				
Referral to Respiratory Specialist	58 (41.7%)	40 (47%)	15 (34%)	3 (30%)
Average time [and range] from pneumonitis diagnosis to respiratory review (days)	34 [0–215]	30 [0–135]	35 [2–128]	75 [2–215]
Broncho-alveolar lavage	33 (23.7%)	28 (32.9%)	5 (11.4%)	0
Average time [and range] from pneumonitis diagnosis to BAL (days)	35 [1–149]	32 [1–149]	52 [6–128]	NA
Pulmonary function test (PFT) following diagnosis of pneumonitis	12 (8.6%)	9 (10.6%)	3 (6.8%)	0
Admission to hospital	54 (38.8%)	39 (45.9%)	13 (29.5%)	2 (20%)
Average duration [and range] of hospital admission (days)	10.5 [1–43]	10 [1–43]	15 [1–39]	2 [1–2]
Admission to CCU/ITU	10 (7.2%)	7 (8.2%)	3 (6.8%)	0
Average duration [and range] of CCU/ITU admission (days)	6.5 [1–20]	7 [1–20]	7 [3–11]	NA

Fifty-eight cases (42%) were referred for specialist respiratory management. The average time from first presentation with pneumonitis to being seen by the respiratory service was 34 days (range 0–215) with 40 and 55% of cases seen within 2 and 3 weeks of pneumonitis diagnosis, respectively. Thirty-three cases (24%) went on to have a bronchoalveolar lavage (BAL). The average time from first presentation with pneumonitis to BAL was 35 days (range 1–149), with 58% of cases having BAL within 3 weeks. For those with grade 2 severity (*n* = 20), average time to BAL was 41 days (range 1–149), and for those with grade 3–5 severity, was 24 days (range 1–91). Twelve cases (8.7%) had documented evidence of pulmonary function tests (PFTs) following diagnosis with pneumonitis.

Fifty-four cases were admitted to hospital with 10 requiring admission to critical care (CCU). The average length of hospital and CCU admission was 10.5 days (range 1–43) and 6.5 days (range 1–20), respectively.

### ICI Related Cases

Of the 85 cases attributed to ICI, 32 (37.6%) had received pembrolizumab, 20 (23.5%) had received nivolumab and 11 (12.9%) combination therapy with ipilimumab and nivolumab (Supplementary Table 2). Thirteen (15.3%) were considered to have superadded infection. Three cases (3.5%) were due to a flare of underlying fibrosis or severe sarcoid-like reaction from ICI.

The mean time from starting ICI therapy to first presentation with pneumonitis was 4.5 months (range 4 days −21 months). Forty-eight percent of cases presented within 3 months.

In 4 cases, patients had completed ICI therapy at the time of presenting with pneumonitis. Of the remaining 81 cases, 28 (34.6%) were re-challenged with ICI therapy. The mean lost treatment time between ICI being held and reintroduced was 73 days (range 11–275), however in some cases this was due to other toxicities e.g., ICI-mediated colitis. Of the 28 cases that were re-challenged, 6 (21.4%) had recurrence of pneumonitis and had to stop treatment. In 6 cases (21.4%), patients had disease progression within 2 cycles after resuming ICI and then stopped treatment. For these 6 cases, the average treatment interruption was 74 days (range 29–150).

A total of 1,565 patients were treated with ICI therapy in our centre between 2015 and 2020. Of these, we identified 85 (5.43%) that developed symptomatic ICI-related pneumonitis (N.B. the 10 patients with pneumonitis considered to be due to both RT and ICI therapy are not described here). Table 3 describes the proportion of pneumonitis cases by tumour-group, the highest of which were head and neck and lung cancers. Figure 2 describes the number of ICI-pneumonitis cases by severity. Of the total number of patients treated with ICI therapy, 62 (3.96%) were Grade 2, 11 (0.7%) were Grade 3, 4 (0.26%) were Grade 4 and 8 (0.51%) were Grade 5, respectively.

**Table 3:** The number of patients receiving ICI therapy and developing symptomatic ICI-related pneumonitis between 2015 and 2020.

**Tumour group**	**Patients receiving** **ICI therapy** **(*n* = 1,565)**	**G≥2 ICI** **pneumonitis cases** **(*n* = 85)**	**Proportion of** **pneumonitis cases** **by tumour** **group (%)**
Breast	35	–	–
GI	144	10	6.9
Gynae	47	–	–
H&N	61	7	11.5
Lung	433	39	9.0
Lymphoma	25	–	–
Sarcoma	29	2	6.9
Skin	522	18	3.4
Urology	269	9	3.3

### RT Related Cases

Forty-four cases were attributed to RT, of which 8 (18%) had received palliative dose-fractionation schedules (ranging from 20 Gy in 5 fractions to 39 Gy in 13 fractions). The mean time from first fraction of radiotherapy to first presentation with pneumonitis was 3.6 months (range 8 days−8 months). Nine cases (20.5%) were considered to have superadded infection. Of the total number treated with RT, 35 (79.5%) were Grade 2, 4 (9.1%) were Grade 3 and 5 (11.4%) were Grade 5, including 2 patients (4.5%) that had underlying fibrosis thought to be exacerbated by RT. The number of RT related cases were stable at approximately 10 per year between 2015 and 2020 (Figure 3).

### Mixed RT and ICI Related Cases

Ten cases were patients with a diagnosis of non-small cell lung cancer (NSCLC) who had received both RT and ICI prior to presenting with pneumonitis. All 10 cases were of grade 2 severity. Of these, 6 patients (60%) received Durvalumab. Five had prior treatment with concurrent chemoradiotherapy (60–66 Gy in 30–33 fractions) and one received Durvalumab after sequential chemoradiotherapy 55 Gy in 20 fractions). The remaining patients were treated with Pembrolizumab (3 cases) or Atezolizumab (1 case) and palliative dose fractionation schedules (ranging from 20 Gy in 5 fractions to 36 Gy in 12 fractions). Three cases (30%) were considered to have superadded infection.

### Healthcare Resource Utilisation

**Figure 4:**
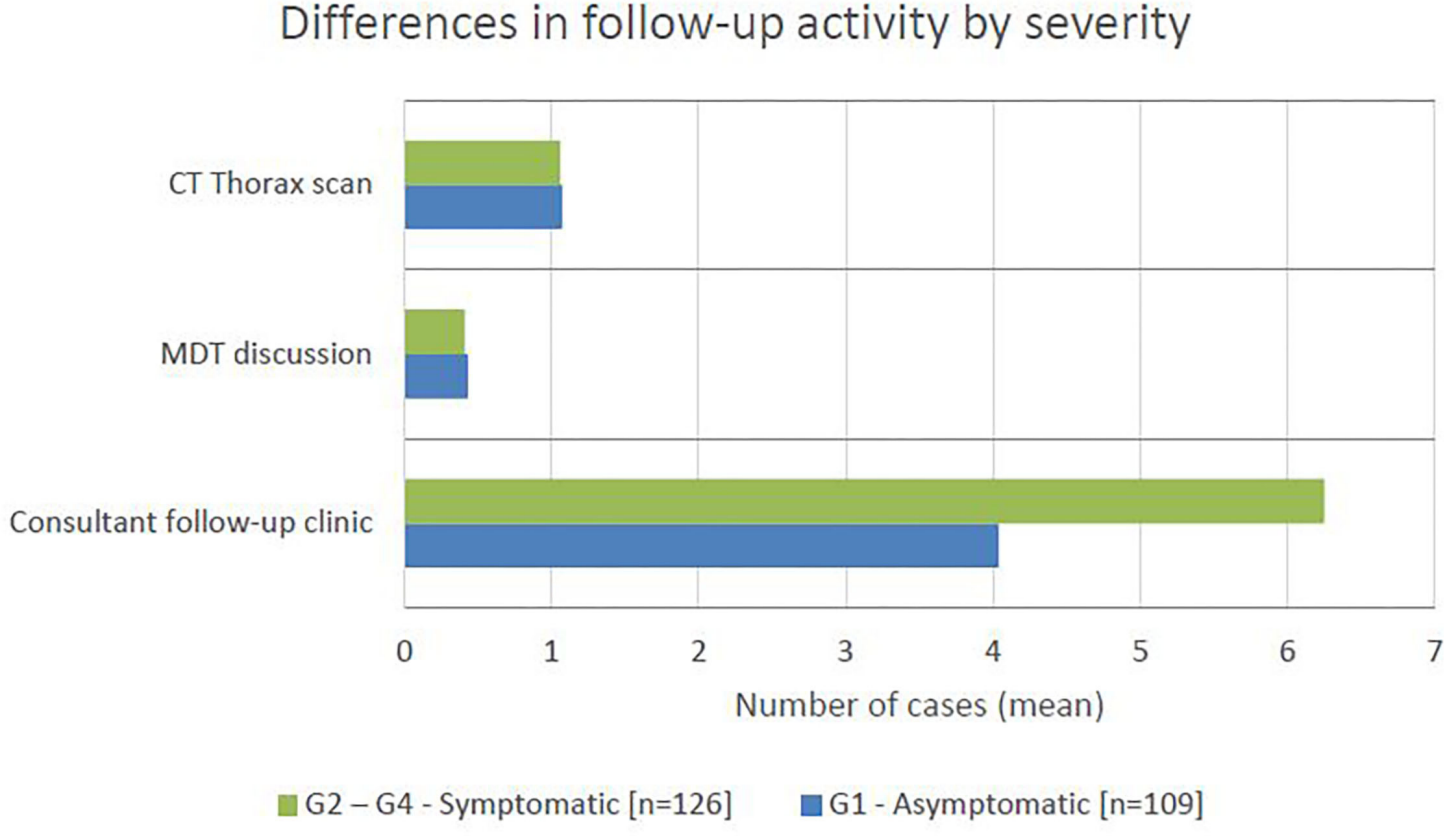
The mean number of cases requiring CT thorax scan, MDT discussion, and consultant follow-up clinic appointment in the 16 weeks following pneumonitis diagnosis, by severity.

The number of consultant follow-up clinic, CT thorax appointments and MDT discussions in the 16 weeks following pneumonitis diagnosis was compared between patients with grade 1 and grade 2–4 severity (Figure 4). The number of CT thorax scans and MDT discussions were broadly equal across the groups, however patients with grade 2–4 severity had approximately 50% more consultant follow-up clinic appointments (unpaired *t*-test: *P* < 0.0001, difference in mean = 2.21, CI 1.34–3.07). Of the patients with grade 2–5 pneumonitis, 54 (39%) were admitted to hospital and 10 (7%) to CCU. The average duration of hospital admission was 10.5 days (range 1–43) per patient and CCU admission was 6.5 days (range 1–20) per patient.

In Table 4, we present the average use of resources associated with managing a symptomatic pneumonitis case. We recognise that resource utilisation will vary depending on severity and not every resource will be used for every case. Estimates for units used are based on data from this study (indicated with ^*^) and expert opinion (indicated with ^∧^) on additional resources that gold-standard care is expected to include (PFTs and follow-up consultation for all cases requiring referral for specialist respiratory opinion). Units used have been weighted based on study data on the expected average use of resources by patients. For example, based on our data, only 4.3% of cases required other immunosuppressive therapy, only 41.7% a respiratory referral, and 23.7% bronchoscopy. Costs per unit were derived from the National Schedule of NHS costs 2019/2020 (NHS reference costs) (10) and British National Formulary (BNF) (11) and allow estimates on the average costs of healthcare per symptomatic pneumonitis case to be made (£3,932.33).

**Table 4:** The estimated average per-patient cost of symptomatic pneumonitis based on NHS reference and BNF unit costs.

**Resource**	**Units used** **(weighted)**	**Cost per** **unit (£)**	**Currency or service code**	**Total estimated** **cost (£)**
Initial respiratory appointment (CL)^*^	0.417	198.52	WF01B	82.78
FU respiratory appointment (CL)^∧^	0.417	209.12	WF02A	87.20
Bronchoscopy^*^	0.237	703.86	DZ69A (OPROC)	166.81
PFT^∧^	0.417	156.75	DZ52Z (OPROC)	65.36
CT Thorax scan^*^	1	68.92	RD21A (IMAG, outpatient)	68.92
MDT discussion^*^	1	127.45	Average of CMDT-B, -C, -LG, -OTH, -SPU, -SPG	127.45
FU Oncology appointment (CL) ^*^	2	200.38	WF01A	400.76
Hospital admission (nights) ^*^	10.5 × 0.07	473.97	DZ19M (NES)	1,940.91
CCU admission (nights)^*^	6.5 × 0.39	933.51	CCU03, XC06Z (CC)	424.75
Other immunosuppressive agent^*^	0.043	13,195	BNF	567.39
Total average cost per patient				£3,932.33

*FU, follow up; CL, consultant led; OPROC, outpatient procedures; IMAG, Diagnostic Imaging; CMDT, Cancer multidisciplinary team meetings; CC, Critical Care; NES, Non-elective short stay. Resource utilisation units have been based on data extracted from healthcare records*

(*) *or expert opinion*

(∧) * and have been weighted based on study data on the expected average use of resources. Infliximab is used here as an example of “other immunosuppressive agent.” The cost per unit is based on a course of 5 mg/kg twice daily for 5 days. For a 70 kg patient this is 3,500 mg. The BNF cost is £377 for 100 mg*.

This is a conservative estimate as we have not included costs for empirical antibiotics, steroids, co-trimoxazole, pathology and microbiology services and have accounted only for CT thorax imaging, whereas in practice patients are likely to have had CT thorax, abdomen, and pelvis imaging. Furthermore, we have assumed patients that were referred for specialist respiratory input had only one initial and one follow-up appointment whereas in practice the number of follow-up appointments may have been higher.

To put this into context, applying the average cost per patient to the 139 symptomatic cases in our study who were treated at our centre between 2015 and 2020 leads to a total cost of £546,594.52. This is a cost of £91,099.09 per year.

## Discussion

In this study, we present new real-world data on the incidence, severity, management, and resource burden of cancer therapy pneumonitis. To our knowledge, this is the first study to look at real-world incidence and healthcare resource utilisation at the per-patient level in a UK cancer hospital. The latter is an important contribution, providing useful health economics data to inform future studies assessing cost-effectiveness of pneumonitis-related healthcare pathways. Additionally, we contribute evidence to the literature on the utility of informatics tools to extract real-world data from the EHR with reduced need for clinician time to manually collect such data.

The grade 2 or higher ICI-pneumonitis incidence in our cohort is 5.43%, which is greater than the all-grade 1.3–2.7% incidence reported in the literature (4, 5) and higher than the all-grade pneumonitis from a large real-world series by Naidoo et al. (3). For example, a meta-analysis of PD-1 inhibitor pneumonitis by Nishino et al. demonstrated an all-grade incidence of 2.7% (5). Whilst our study also included PDL-1 and CTLA-4 related pneumonitis, making direct comparison difficult, this value is much lower than seen in our cohort. Similarly, grade 3–5 incidence in our cohort was 1.49%, higher than the 0.8% Grade 3–5 PD-1 pneumonitis rate described by Nishino et al. (5). Time to onset of ICI-pneumonitis was also longer in our cohort (mean 4.5 months, range 4 days-21 months), compared to the median 2.7 months (range 9 days−19.2 months) described by Naidoo et al. (3). In 91 patients receiving adjuvant Durvalumab following RT for NSCLC at our centre over the study period, 6 (6.59%) developed pneumonitis, all of which were grade 2 severity. Our data demonstrates lower rates of grade 3–4 pneumonitis than the PACIFIC study, which reported all-grade and grade 3–4 pneumonitis rates of 12.6 and 1.9%, respectively.

The incidence of pneumonitis in patients treated with combination ipilimumab and nivolumab was 3.75%, lower compared to the incidence from single agent ICI. This contrasts findings in the literature which describe ICI-related pneumonitis most commonly occurring in patients receiving combination treatment (3, 8). A higher incidence of ICI-pneumonitis is reported in patients with non-small cell lung cancer and with squamous cancers (5, 8). This is reflected in our cohort which demonstrated highest incidence in head and neck (11.48%) and lung (9.01%) tumour groups.

Twenty-eight patients who developed ICI-pneumonitis at our centre had ICI re-introduced on resolution of symptoms. Six (21.4%) had disease progression within 2 cycles of restarting treatment. The average interruption in treatment was 74 days (range 29–150) and it is possible that this interruption may have led to loss of immunomodulation and control of cancer.

Almost 20% of RT pneumonitis cases had received palliative dose-fractionation schedules. Whilst there may be confounding factors including larger irradiated volume and possibly frailer patients with heart-failure or disease progression contributing to the presentation, this may be an important factor when considering quality of life in deciding which patients would benefit from palliative treatment. Mean time to onset of RT pneumonitis in our cohort fit with that described in the literature (6).

Of note, the number of cases of ICI-pneumonitis increased year on year from 2015 to 2019 but then reduced in 2020. This is inconsistent with the number of patients treated with ICI overall at our centre which continued to increase in 2020 (Supplementary Table 3). The explanation for this is likely multifactorial, however one possibility may be the impact of the COVID-19 pandemic, due to increased local hospital care or misdiagnosis of ICI-pneumonitis as COVID-19 due to overlapping clinical and radiological features (12–14), particularly if patients presented to departments less familiar with the diagnosis and management of ICI-toxicity. It is possible that growing understanding of ICI-pneumonitis results in earlier identification and optimal management. In particular, there is a need to identify patients at risk of developing grade ≥3 pneumonitis as early as possible and this is an area where artificial intelligence and machine learning predictive models may provide significant clinical utility.

Such considerations support the need for wider education on cancer therapy toxicity including differential diagnoses and the potential role of acute oncology, respiratory and microbiology services in recognising this. Continued surveillance and analysis of pneumonitis incidence will be important and may be benefited by a national pneumonitis registry. Here we have provided an indicative list of resource use for symptomatic pneumonitis based on a combination of study data and expert opinion, and an indicative, conservative estimate of healthcare cost per patient. Whilst we do not have data on the UK-wide incidence for ICI or radiotherapy-pneumonitis to provide estimates at population-level, the economic impact at our centre is estimated at over £90,000 per year. Earlier detection and improved management of pneumonitis could potentially reduce such costs. Future research on the health economic aspects of ICI or radiotherapy-pneumonitis will allow us to estimate more precisely the economic burden on the NHS.

Our study has several limitations. Due to its retrospective nature, the data presented relies on EHR documentation which may contain inaccuracies, especially when referring to investigations performed outside our centre such as BAL and PFTs. An example was concurrent administration of prophylactic co-trimoxazole with steroids which appeared variable. It is difficult to ascertain from retrospective EHR data the reasons for this. One explanation is that steroids were started on the assumption that symptoms would improve and when they didn't, the course was extended and prophylactic co-trimoxazole was introduced. Improved education on recording key clinical decisions in medical notes is often encouraged as a gold-standard of documentation. Considering informatics requirements with respect to such principles highlights a need for more structured data fields that in-turn could integrate with drug toxicity analytics and decision support systems—e.g., the recording of a steroid prescription in the EHR then presents the clinician with a data field asking if prophylactic co-trimoxazole, proton-pump inhibitor or bisphosphonate is indicated, thus providing additional data at the same time as standardising care.

A further limitation of informatics approaches is that the identification of cases is dependent on defining comprehensive search terms. Missed cases, or indeed those investigated with chest x-ray rather than CT would lead to underestimates of the incidence of pneumonitis in this cohort or favour identification of milder cases that are defined with radiological reports but not actioned by clinical teams as significant. Informatics approaches do however tend to be more comprehensive than manual searches for cases given their broad vision of the entire EHR dataset and are therefore a valuable route to define descriptive statistics of the broader cohort identified, which may improve upon more traditional methods of audit and research and can be scaled up to larger data sets, with less person-hour requirements.

This is a single-centre study and therefore may not reflect variation in practice across centres, but our experience is that such tools can be readily applied in other centres and are thus well-placed to drive acquisition of much larger datasets, in collaboration with others, where this can facilitate greater understanding and allow comparison between centres or creation of a lung toxicity registry.

Overall, the use of ICI has increased over the last 6 years and with that as has the incidence of ICI-related pneumonitis. Our data suggests the incidence of ICI-pneumonitis is higher, and time to onset is longer compared to the literature. The number of patients receiving ICI therapy and subsequently the number presenting with possible ICI-pneumonitis is likely to continue to increase, placing demand on acute oncology and specialist respiratory services, and potentially hospital and CCU admissions. It is imperative that funding and resource provision is made available to support increasing demand on such pathways and to ensure adequate provision of acute oncology, respiratory and microbiology services—for example, rapid access to BAL to aid diagnosis, and PFTs to define severity. Improved management of pneumonitis may facilitate prompt continuation of cancer therapy, and improved outcomes for this not insubstantial proportion of our ICI or thoracic radiotherapy treated patients.

## Data Availability Statement

The original contributions presented in the study are included in the article/Supplementary Material, further inquiries can be directed to the corresponding author/s.

## Author Contributions

RL: guarantor of integrity of the entire study. SH, DC, MA, BS, KG, PM, NY, and RL: study concepts and design. SH: literature research and manuscript preparation. SH, DC, KG, BH, HK, and RL: data analysis. SH, KG, BH, MA, HK, BS, PM, WC, MD, NY, JB, AM, IL, FM, MO'B, SP, and RL: manuscript editing. All authors contributed to the article and approved the submitted version.

## Author Disclaimer

The views expressed are those of the author(s) and not necessarily those of the NIHR or the Department of Health and Social Care.

## Conflict of Interest

SH is funded by the UKRI CDT in AI for Healthcare http://ai4health.io (Grant No. P/S023283/1), by Imperial College London and by the Royal Marsden & Institute of Cancer Research NIHR Biomedical Research Centre. KG is supported by the National Institute for Health Research (NIHR) Biomedical Research Centre at The Royal Marsden NHS Foundation Trust and the Institute of Cancer Research, London. WC receives grant funding from Breast Cancer Trials, funding from Australian Government Research Training scholarship and honoraria from Janssen and AstraZeneca, outside the submitted work. BH is funded by Cancer Research UK and Royal Marsden Partners. HK is funded by the Royal Marsden Cancer Charity. PM is supported by the Action for Pulmonary Fibrosis Mike Bray Fellowship. FM reports speaker fees from Astra Zeneca, Elekta, and Takeda; research grant funding from MSD and consulting fees from Astra Zeneca and Accuray, outside of the submitted work. MO'B reports advisory work for MSD, BI, Abbot, Pierre Fabre, and Roche outside the submitted work. SP reports personal fees from BMS, Roche, Takeda, AstraZeneca, Pfizer, MSD, EMD Serono, Guardant Health, Abbvie, Boehringer Ingelheim, OncLive, Medscape, Incyte, Paradox Pharmaceuticals, Eli Lilly, outside the submitted work. RL is funded by the Royal Marsden Cancer Charity with grant funding from Cancer Research UK, Innovate UK (co-funded with Roche and Optellum), and RM Partners outside of the submitted work. The remaining authors declare that the research was conducted in the absence of any commercial or financial relationships that could be construed as a potential conflict of interest.

## Publisher's Note

All claims expressed in this article are solely those of the authors and do not necessarily represent those of their affiliated organizations, or those of the publisher, the editors and the reviewers. Any product that may be evaluated in this article, or claim that may be made by its manufacturer, is not guaranteed or endorsed by the publisher.
