# Supplementary material for: Immune Checkpoint Inhibitor and Radiotherapy-Related Pneumonitis: An Informatics Approach to Determine Real-World Incidence, Severity, Management, and Resource Implications
Source: Front Med (Lausanne). 2021 Nov 1;8:764563. doi: 10.3389/fmed.2021.764563 (PMC8591134; doi:10.3389/fmed.2021.764563)
Supplement: Supplementary file 1 [file Table_1.DOCX]

**Supplementary Material**

**Supplementary Table 1: CTCAE v5.0 Pneumonitis severity score.**

| **CTCAE Grade** | **Severity based on retrospective case-note review** |
| --- | --- |
| 1 | Asymptomatic, intervention not indicated |
| 2 | Symptomatic, medical intervention (e.g. antibiotics/steroids) indicated |
| 3 | Severe symptoms, oxygen indicated |
| 4 | Life-threatening, urgent intervention (e.g. intubation) indicated |
| 5 | Death |

**Supplementary Table 2: Number of symptomatic ICI pneumonitis cases by drug.**

| **ICI drug therapy** | **Number of cases of G2-5 pneumonitis** | **Total number of patients treated** | **G2-5 pneumonitis rate (%)** |
| --- | --- | --- | --- |
| Ipilimumab/Nivolumab combination | 11 | 293 | 3.75 |
| Ipilimumab | 1 | 1 | 100.00 |
| Nivolumab | 20 | 334 | 5.99 |
| Atezolizumab | 12 | 142 | 8.45 |
| Avelumab | 3 | 64 | 4.69 |
| Pembrolizumab | 32 | 640 | 5.00 |
| Durvalumab | 6 | 91 | 6.59 |
| TOTAL | 85 | 1565 | 5.43 |

**Supplementary Table 3: Number of patients commenced on ICI per year.**

| **Year** | **Number of patients commenced on ICI drug** |
| --- | --- |
| 2015 | 39 |
| 2016 | 127 |
| 2017 | 158 |
| 2018 | 270 |
| 2019 | 477 |
| 2020 | 494 |
